# SARS Transmission Pattern in Singapore Reassessed by Viral Sequence Variation Analysis

**DOI:** 10.1371/journal.pmed.0020043

**Published:** 2005-02-22

**Authors:** Jianjun Liu, Siew Lan Lim, Yijun Ruan, Ai Ee Ling, Lisa F. P Ng, Christian Drosten, Edison T Liu, Lawrence W Stanton, Martin L Hibberd

**Affiliations:** **1**Genome Institute of SingaporeSingaporeSingapore; **2**Department of Pathology, Singapore General HospitalSingapore; **3**Bernhard Nocht Institute for Tropical Medicine, National Reference Center for Tropical Infectious DiseasesHamburgGermany; Sunnybrook and Women's College Health Sciences CentreCanada

## Abstract

**Background:**

Epidemiological investigations of infectious disease are mainly dependent on indirect contact information and only occasionally assisted by characterization of pathogen sequence variation from clinical isolates. Direct sequence analysis of the pathogen, particularly at a population level, is generally thought to be too cumbersome, technically difficult, and expensive. We present here a novel application of mass spectrometry (MS)–based technology in characterizing viral sequence variations that overcomes these problems, and we apply it retrospectively to the severe acute respiratory syndrome (SARS) outbreak in Singapore.

**Methods and Findings:**

The success rate of the MS-based analysis for detecting SARS coronavirus (SARS-CoV) sequence variations was determined to be 95% with 75 copies of viral RNA per reaction, which is sufficient to directly analyze both clinical and cultured samples. Analysis of 13 SARS-CoV isolates from the different stages of the Singapore outbreak identified nine sequence variations that could define the molecular relationship between them and pointed to a new, previously unidentified, primary route of introduction of SARS-CoV into the Singapore population. Our direct determination of viral sequence variation from a clinical sample also clarified an unresolved epidemiological link regarding the acquisition of SARS in a German patient. We were also able to detect heterogeneous viral sequences in primary lung tissues, suggesting a possible coevolution of quasispecies of virus within a single host.

**Conclusion:**

This study has further demonstrated the importance of improving clinical and epidemiological studies of pathogen transmission through the use of genetic analysis and has revealed the MS-based analysis to be a sensitive and accurate method for characterizing SARS-CoV genetic variations in clinical samples. We suggest that this approach should be used routinely during outbreaks of a wide variety of agents, in order to allow the most effective control.

## Abstract 2

Mass spectrometry-based sequence analysis provides a sensitive and accurate method to characterize genetic variation of the SARS coronavirus in clinical samples

## Introduction

During infectious disease outbreaks, due to either new or established agents, extensive information gathering is required to enable identification of the source, transmission routes, and the effect of containment policies. It is becoming increasingly clear that traditional approaches based on travel and contact tracing are not sufficient for tracking an outbreak. New sequence-based techniques for pathogen detection and identification have the potential to become perhaps the most important component of these investigations, as demonstrated in the recent worldwide effort in fighting the epidemic of severe acute respiratory syndrome (SARS). The discovery of the SARS coronavirus (SARS-CoV) as the etiological agent for SARS was a major breakthrough [1], which was quickly followed by the successful sequencing of the whole genome of the virus [2,3]. Genome sequence comparison between this new coronavirus and the three known classes of coronavirus revealed a similar genome structure, but minimum homology at the amino acid level, strongly suggesting that the SARS-CoV was a new class of coronavirus [2,3,4]. A comparative sequence analysis of 14 SARS isolates from different countries suggested a moderate genetic diversity among the SARS isolates and thus implied a slow evolution of the SARS-CoV genome [5]. Furthermore, sequence variation analyses of SARS-CoV isolates demonstrated that common genetic variations in the SARS-CoV genome could be used as “molecular fingerprints” to partition the viral isolates into different genetic lineages, track the transmission of a specific viral lineage, and infer the origin of infection [5,6]. Therefore, the characterization of SARS-CoV's genetic variations is not only instrumental for understanding its genetic diversity and genome evolution but also important for tracking its transmission and understanding its epidemiological pattern in human populations.

Direct sequence analysis of a pathogen in a large number of clinical samples, particularly at a population level, is generally cumbersome, technically challenging, and expensive. Therefore, a rapid, sensitive, high-throughput, and cost-effective screening method would greatly facilitate large-scale characterization of genetic variation of pathogens at a population level. A mass spectrometry (MS)–based method for detecting single nucleotide polymorphisms has been routinely used as a high-throughput method for genotyping human samples, and, thus, we sought to extend this methodology to detect pathogen sequence variations. Here, we demonstrate the high sensitivity of the MS-based analysis in detecting SARS-CoV sequence variations and apply it to analyzing both cultured viral isolates and uncultured tissue samples of SARS-CoV.

## Methods

### Patients and Samples

The SARS-CoV samples used in the sensitivity study were previously described [7]. Briefly, five in vitro samples were generated by spiking 200-μl human whole-blood samples with SARS-CoV virus obtained from a Vero E6 cell culture of an anonymous Singapore patient.

Vero cell cultured viral isolates were obtained from 13 Singapore patients: the presumed index case (patient Sin2500) of the Singapore SARS outbreak, whose date of illness onset was 25 February 2004; three primary contacts (patients Sin2677, Sin2774, and Sin2748), whose dates of onset were 9 March 2004 (Sin2677 and Sin2774) and 14 March 2004 (Sin2748); one secondary contact (patient Sin2679), who was believed to have contracted SARS from index patient Sin2500 through another primary contact not included in this study and whose date of illness onset was 15 March 2004; and another eight patients (Sin842, Sin845, Sin846, Sin847, Sin848, Sin849, Sin850, and Sin852), who were believed to be the fifth- or six-generation contacts of index patient Sin2500 (based on contact tracing records) and whose dates of illness onset ranged from 2 April to 14 April 2004. All the patients fitted the World Health Organization case definition for probable SARS [8]. The virus was cultured in vero cells following isolation from respiratory samples (three endotracheal tube swabs, three throat swabs, one nasal swab, two nasopharyngeal aspirates, and four lung tissues) obtained from the patients between 0 and 11 d after onset of symptoms. Uncultured lung tissue samples were also obtained from patients Sin842, Sin848, Sin849, and Sin852. Bronchoalveolar lavage material was obtained from a serologically confirmed German patient with SARS who had been traveling on the same flight as an early Singaporean patient with SARS who was later hospitalized in Germany [9]. Virus could not be isolated from the sample owing to its inappropriate storage.

### RNA Extraction and cDNA Synthesis

For the spiked human blood samples, RNA was extracted from 200 μl of blood into 50 μl of water using a HighPure RNA kit (Roche, Basel, Switzerland). For the clinical samples, RNA was extracted into 30 μl of water using a QiAmp viral RNA mini kit (Qiagen, Valencia, California, United States). RNA samples were reverse transcribed into cDNA using 2 μl of RNA as template, a SuperScript kit (Invitrogen, Carlsbad, California, United States), and 13 sequence-specific primers [10]. All cDNA products were purified by ethanol precipitation and then resuspended in 20 μl of water.

### Real-Time Quantitative PCR Analysis

Real-time quantitative PCR analyses of the RNA samples from spiked human blood were performed using a Lightcycler Sars-CoV quantification kit (Roche). Each analysis was done using 1 μl of RNA and in accordance with the manufacturers' instructions.

### Single Nucleotide Variations of SARS-CoV

Twenty-one single nucleotide variations (SNVs) of SARS-CoV were analyzed in this study. Eight of these were identified from our previous sequence analysis of five Singapore SARS-CoV viral isolates and represent variations [5], and 13 SNVs were identified from a more recent genome sequence analysis of additional Singapore SARS-CoV viral isolates. The former eight SNVs were used in evaluating the sensitivity of MS-based genotyping analysis in detecting SARS-CoV viral genotypes, and the later 13 SNVs, as well as five of the former eight SNVs, were used to genotype the patient-derived samples.

### SNV Analysis

A primer extension genotyping assay was designed for each SARS-CoV SNV using SpectroDesigner software (Sequenom, San Diego, California, United States) and analyzed using the MassARRAY system (Sequenom) and the recommended protocol for the MADLI-TOF (matrix-assisted laser desorption ionization/time-of-flight) MS-based genotyping analysis [11]. One microliter of cDNA (equivalent to 0.1 μl of RNA from 1 μl of spiked human blood) was used as template in each analysis.

### Statistical Analysis of Sensitivity

The analytical detection limit of the combined RNA preparation/RT-PCR/MALDI-TOF MS system was determined by probit analysis [12] using the Statgraphics Plus 5.0 software package (Statistical Graphics, Jena, Germany).

## Results

### Sensitivity for Detecting SARS-CoV SNVs

Prior to determining viral sequence variants in clinical samples, we measured the sensitivity of the MS-based assay for detecting SARS-CoV sequence variations by analyzing eight SNVs in in vitro human blood samples spiked with SARS-CoV. The viral RNA copy numbers in five spiked human blood samples were quantified by real-time PCR analysis and determined to be 1.64 × 10^6^ (SB1), 3.84 × 10^3^ (SB2), 2.17 × 10^3^ (SB3), 6.21 × 10^2^ (SB4), and 1.20 × 10^2^ (SB5) copies per microliter (Figure S1). The three samples (SB3, SB4, and SB5) with the lowest virus copy numbers were used to determine the sensitivity of the MS-based assay.

We were able to successfully type the virus in 16 of the 16 analyses of the SB3 sample (equivalent to 217 RNA copies per reaction), 12 of the 15 analyses of the SB4 sample (equivalent to 62 RNA copies per reaction), and six of the 15 analyses of the SB5 sample (equivalent to 12 RNA copies per reaction). According to probit analysis, this corresponds to a 95% probability of detection when at least 75 copies of viral RNA (95% confidence interval, 59–107 copies) are present per reaction, and still a 50% detection chance at 38 copies of virus RNA per reaction (95% confidence interval, 29–53 copies). All 64 sham spiked samples gave negative genotype calls.

### Sequence Variant Determination of SARS-CoV in Viral Isolates

Having demonstrated the high sensitivity of the MS-based analysis, we typed 18 SARS-CoV SNVs in the cultured viral isolates from nine Singapore patients, including five early Singapore cases (Sin2500, Sin2774, Sin2748, Sin2677, and Sin2679) and four later Singapore cases (Sin842, Sin848, Sin849, and Sin852).

**Figure 1:**
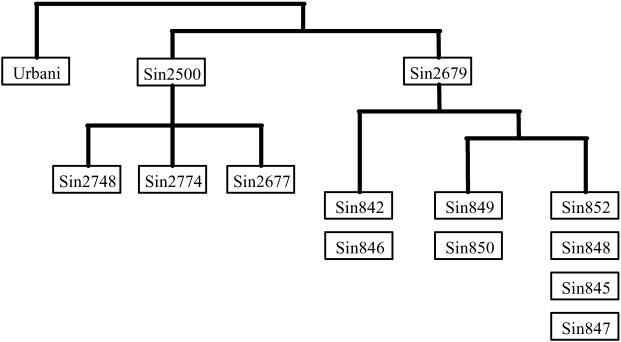
The Molecular Relationship among 13 Singapore SARS-CoV Isolates Based on the Genotype Sharing Pattern of the Viral Isolates

Of the 18 SNVs analyzed, nine were detected in at least two SARS isolates (Table 1). Assuming that a common sequence variation originated through a single mutation in a host and was then propagated by subsequent infection to others, the sharing of a specific sequence variant by different viral isolates suggests that either these viral isolates share a common ancestor or they have direct ancestor–descendant relationship. Sequence variants at the nine common SNV sites were used to reconstruct the molecular relationship among the nine viral isolates. The pattern of the shared variants among the nine viral isolates (Table 1) clearly indicated two major molecular lineages of isolate (Figure 1). One lineage includes the four early isolates from patients Sin2500, Sin2774, Sin2748, and Sin2677, and the other includes the early isolate from patient Sin2679 and the four later isolates. The first lineage is defined by the sequence variant T:C:T at SNV positions19,084, 23,174, and 28,268, whereas the second lineage is defined by the sequence variant C:T:C. Both the variants are distinct from the presumed ancestral sequence variant C:C:C observed in the Urbani viral isolate. The later Singapore isolates were also differentiated from the early isolates by the sequence variant pattern at SNV positions 22,549 and 23,735.

**Table 1:**
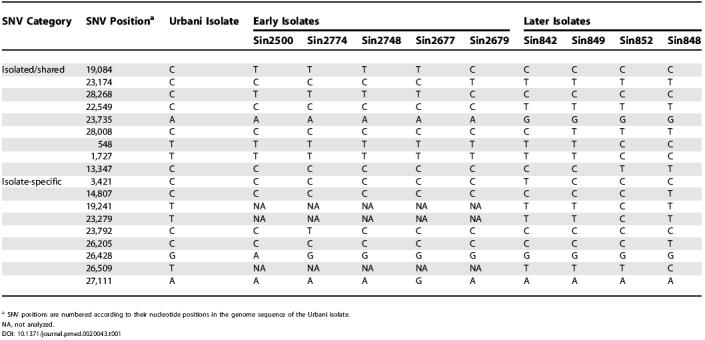
Genotypes of Five Early and Four Later Singapore SARS-CoV Isolates in 18 SNVs

^a^ SNV positions are numbered according to their nucleotide positions in the genome sequence of the Urbani isolate

NA, not analyzed

In addition, the sequence variant sharing pattern at SNVs 28,008, 548, 1,727, and 13,347 can further differentiate the four fifth- and six-generation isolates into three different sub-lineages (Figure 1) defined by three distinct variants: C:T:T:C in the isolate from patient Sin842, T:T:T:C in the isolate from patient Sin849, and T:C:C:T in the isolates from patients Sin852 and Sin848 (Table 1). To further confirm this three-sub-lineage pattern observed in the later Singapore isolates, we typed another four later Singapore isolates, from patients Sin845, Sin846, Sin847, and Sin850, at five critical SNVs (19,084, 28,008, 548, 1,727, and 13,347). The detected sequence variations in the four new isolates supported the three-sub-lineage pattern in the later Singapore isolates (Table 2; Figure 1). The second and third sub-lineages are more closely related to each other than to the first one, as all the members of the latter two sub-lineages show the variant T at SNV position 28,008, whereas the two members of the first sub-lineage show the variant C.

**Table 2:**
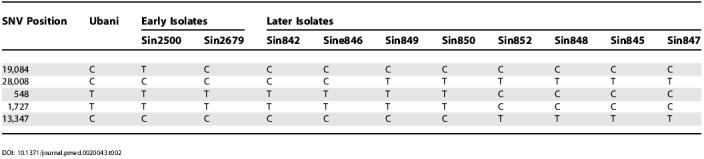
Genotypes of Two Early and Eight Later Singapore SARS-CoV Isolates at Five SNV Positions

The molecular relationship among the viral isolates derived from MS-based viral sequence analysis is consistent with the one derived from the whole-genome sequence analysis of the same isolates [13], clearly demonstrating that a small subset of commonly shared variances can be used as “molecular signature” to differentiate and thus track viral isolates.

### Direct Sequence Variation Determination of SARS-CoV in Primary Lung Tissue Samples

We also typed the 18 SNVs in four uncultured lung tissue samples from patients Sin842, Sin848, Sin849, and Sin852, and compared their sequence variations with their matched cultured isolates (Table 3).

**Table 3:**
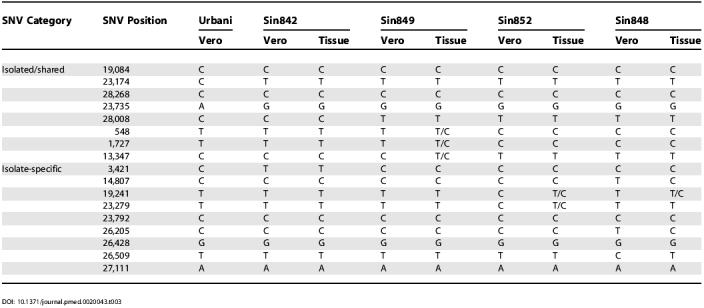
Comparison of Paired Direct Tissue Samples and Vero Cell Cultured Isolates of SARS-CoV

**Figure 2:**
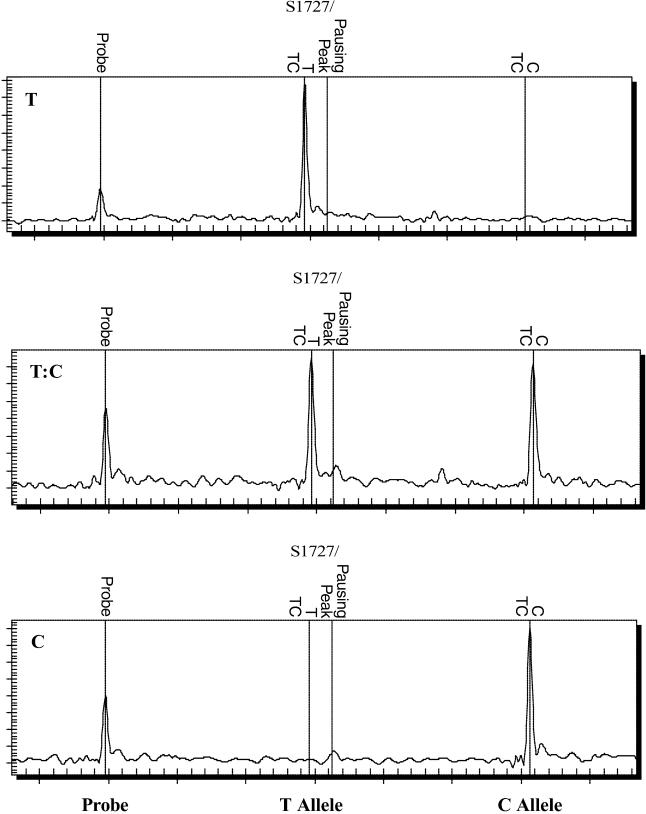
MS Spectrums of the Three Distinct Genotypes at SNV Position 1,727 The T example is from the cultured viral isolate from patient Sin849, the T/C example is from the uncultured lung tissue sample from patient Sin849, and the C example is from the cultured viral isolate from patient Sin852.

Of the 72 direct sequence variation comparisons (18 SNVs in four sample pairs), nine differences were identified. Six of these were due to heterogeneous sequences in primary tissue samples. For example, the cultured isolate from patient Sin849 showed the sequence variant T:T:C at SNV positions 548, 1,727, and 13,347, a subset of the heterogeneous T/C:T/C:T/C variant observed in the matched lung tissue sample from the same patient. Direct comparison of the MS spectrums of the three different sequence variants (T, T/C, and C) at SNV position 1,727 in Figure 2 clearly ruled out the possibility of variant miscall. More interestingly, the heterogeneous variant T/C:T/C:T/C in the primary lung tissue sample revealed both of the two existing variants of T:T:C and C:C:T seen in other cultured and lung tissue samples (Table 3).

In addition, another three sequence variant differences were observed between the paired cultured and tissue samples from patient Sin848 at SNV positions14,807, 26,205, and 26,509, where the tissue sample showed the sequence variant C:C:T, but the matched culture isolate showed the variant T:T:C (Table 3). In all three SNV positions, the cultured isolate showed novel sequence variants, whereas the primary lung tissue showed the Urbani isolate's variants (see Table 3).

### Confirmation of the Singapore Origin of a German SARS-CoV Isolate

The application of tagging and thus tracking of SARS-CoV strains using viral lineage- and/or strain-specific sequence variants was further demonstrated in our investigation of a clinical sample from a German patient. This patient stayed in Hanoi, Vietnam, before sharing an airplane flight with an early Singapore SARS patient on his way to New York via Frankfurt [9]. German health authorities assumed that this patient was infected somewhere in Hanoi, Vietnam, although the possibility of him being infected by a Singapore SARS-CoV strain during his flight to New York could not be ruled out.

We genotyped the virus directly from a brochoalveolar lavage specimen from the German patient at four SNV positions (19,084, 23,792, 26,428, and 27,111) that were distinctive for the early Singapore SARS-CoV isolates (see Table 1). The sequence of the isolate from the German patient was determined to be T:C:G:A at these SNV positions. The detection of the variant T at SNV position 19,084 in the isolate from the German patient strongly suggested that this patient was indeed infected by an early Singapore SARS-CoV strain, as the T sequence variant at position 19,084 was detected only in the early Singapore isolates [5,6] and is clearly different from the C variant at position 19,084 observed in the Vietnam-originated isolates [6,14]. Furthermore, this German isolate's sequence variant, T:C:G:A, was detected only in the isolate from the Singapore primary case Sin2748, suggesting that this German patient was probably infected by a SARS-CoV strain originating from or closely related to the SARS-CoV strain of the Singapore primary case Sin2748 and not from the Hanoi outbreak. Indeed, the T:C:G:A variant is also present in the Frankfurt-1 isolate [15], which was contracted from the early Singaporean patient traveling on the same flight as the German patient.

## Discussion

Through the application of this MS mini-sequencing approach to the SARS outbreak in Singapore we have demonstrated the precision that pathogen sequence data can add to an epidemiological investigation. We analyzed 18 SARS-CoV SNVs and determined the molecular relationship among the viral isolates from 13 Singapore SARS patients (see Figure 1) from different stages of the Singapore outbreak. The molecular relationship among the patients' viral isolates derived from our MS-based viral sequence analysis is not consistent with the current understanding of the clinical transmission relations between these patients. According to contact tracing records, patient Sin2500 was believed to have been the index case of the Singapore SARS outbreak and to have introduced the SARS-CoV virus into the Singapore population following a visit to the Hotel M (Hong Kong) [5,6]. Patients Sin2774, 2748, and Sin2677 were believed to have been infected directly by the index case Sin2500, and patient Sin2679 was believed to have been infected by the index case through another, unidentified, primary patient [5]. Given the pattern of sequence variations observed in the viral isolates, in order for this presumed clinical transmission relationship among these five patients to be correct, one has to assume that during viral transmission from index case Sin2500 to secondary case Sin2679 via an unidentified primary case (two human-to-human transmissions), two reverse mutations at SNV positions 19,084 and 28,268 and one novel mutation at SNV position 23,174 occurred. Although this is not impossible, it is unlikely considering the observed mutation rate among the post–Hotel M SARS-CoV isolates [5,16,17]. A more parsimonious explanation would be a single-mutation scenario in which, instead of contracting the virus from the presumed index case Sin2500, the secondary patient Sin2679 and all the later Singapore cases were infected by a virus strain from the Hotel M cluster through another, as yet unidentified, route, and during this transmission, a novel mutation occurred at SNV position 23,174. Thus, patient Sin2679 or another unidentified Singapore patient from whom patient Sin2679 contracted SARS should be the index case of all the late-generation Singapore SARS patients. Further viral genetic characterization of additional Singapore SARS cases, especially early-generation ones, may shed light on this hypothesis. Unidentified secondary SARS-CoV infection routes from Guangdong to Hong Kong were also suggested by the genetic characterization of SARS-CoV isolates, although none of these contributed substantially to the subsequent Hong Kong outbreak [6,18].

A further application of MS-based viral sequence variation analysis in tracking the virus strain and thus the transmission of SARS-CoV was demonstrated by our confirmation of the Singapore origin of a SARS-CoV isolate from a German patient. Travel and contact tracing records for this German patient indicated more than one potential exposure to SARS-CoV, and because virus could not be cultured from the patient, it was difficult to pinpoint the origin of his infection by classical sequencing methods that require virus enrichment by culture. By genotyping the four SNV positions that showed unique variants in the early Singapore SARS-CoV isolates, we confirmed that this German patient was indeed infected by an early Singapore virus strain, most likely in a hitherto unnoticed aircraft transmission event from an early Singaporean patient who was later hospitalized with SARS in Germany [9]. Therefore, our results clearly demonstrate the usefulness of the sequence variation information as molecular fingerprint in “tagging” SARS-CoV viral strains.

Direct viral sequence variation analysis of uncultured lung tissue samples identified cases of heterogeneous viral sequences in single patient samples. As the SARS-CoV virus is a single-strand RNA virus, the discovery of different sequences in a single tissue sample suggests the presence of multiple viral sequence variants, or quasispsecies, within the host when the sample was retrieved. Our result has further confirmed a recent observation of SARS-CoV quasispecies in individual patients [19] and is consistent with observations in other viral infections. Furthermore, direct comparison between cultured and uncultured samples from the same patient confirmed the existence of the heterogeneous viral sequences only in the uncultured tissue sample, which suggests that of the two initial variants in the human host, only one survived in the vero cell culture. This raises a concern that viral genetic characterization in cultured viral isolates may not capture the whole sequence variation spectrum of a virus in a patient population.

Our study has clearly demonstrated the advantages of MS-based genetic analysis as a method for large-scale viral genetic characterization in clinical samples. Firstly, MS-based analysis has high sensitivity, providing successful detection of virus more than 95% of the time at virus concentrations as low as 75 RNA copies per reaction (equivalent to a detection sensitivity of 10^3^–10^4^ RNA copies per milliliter), which is close to the detection limit of real-time RT-PCR based diagnostic tests (demonstrated to be 5–85 copies of viral RNA per reaction) [7,20,21,22] and within the concentration range reported for SARS-CoV in respiratory and plasma samples [9,20,22,23,24]. Typical RT-PCR sequencing usually requires as many as 1,000 copies of template, as large PCR fragments are typically amplified with less efficiency than the small fragments (about 100 bp) that are commonly used in MS-based analysis. Secondly, our detection of heterogeneous viral sequences in single clinical samples demonstrated the accuracy of the MS-based assay in characterizing SARS-CoV sequence variations. Thirdly, MS-based assay requires only a small amount of starting material for genetic characterization, 0.1 μl of RNA per reaction in the present study. Currently, MS-based genotyping analysis of human genetic variation is routinely done in a multiplex fashion, where multiple single nucleotide polymorphisms are genotyped simultaneously in a single assay. It is thus conceivable that the development of a multiplexing MS-based SNV assay for SARS-CoV could further reduce the required amount of starting material, which would be especially beneficial for analyzing uncultured clinical samples, in which viral materials are often limited. Multiplexing analysis of MS-based assays also greatly reduces the cost of analysis to about US$0.10–$0.20 per analysis (depending on the level of multiplexing), which is much cheaper than conventional sequence analysis, whose cost is typically a few dollars per analysis. Therefore, MS-based sequence variation analysis is a sensitive, accurate, cost-effective, and high-throughput method for confirming putative variations and characterizing known variations in clinical samples, especially for large-scale population studies.

MS-based sequence variation analysis is complementary to the identification of new sequence variation by direct sequence analysis and is particularly suitable for investigating agents for which there is already extensive sequence information. Direct sequence analysis is still the “gold standard” for identifying new sequence variations, but it is inefficient and is not necessary for characterizing known sequence variations in a large number of samples. A combination of initial characterization of genome sequence by direct sequence analysis in a subset of samples and the subsequent analysis of informative genetic variations via a MS-based approach is more efficient and suitable for large-scale population investigations. The genome sequences of a wide variety of pathogens and strains are being rapidly accumulated. In bacterial pathogens, strain sequence information is frequently limited to a relatively small number of genes (where using all of them simultaneously might be appropriate), whereas in viruses such as influenza, extensive genomic knowledge is accumulating. Such accumulation of both partial- and whole-genome sequence information for pathogens will further extend the usefulness of this approach.

In summary, our reassessment of the SARS-CoV transmission route in Singapore using MS-based viral sequence variation analysis highlighted the limitation of conventional epidemiological analysis based on travel and contact tracing, and the importance of informing clinical and epidemiological investigation of pathogen transmission by genetic analysis. With its demonstrated high throughput [25], sensitivity, accuracy, and cost effectiveness in determining viral sequence variations, MS-based genetic analysis can greatly facilitate the large-scale epidemiological investigations of SARS-CoV and other agents of infectious disease, and may allow for real-time investigation in outbreak situations.

## Supporting Information

Figure S1 Detection of SARS-CoV by Real-Time Quantitative PCR in Spiked Human Blood Samples The x-axis denotes the cycle number of the quantitative PCR assay, and the y-axis denotes fluorescence intensity (F2) over the background level. RNA standards were as follows: 1.05 × 10^6^ copies per reaction (line a), 1.01 × 10^5^ copies per reaction (line b), 9.4 × 10^3^ copies per reaction (line c), 8.9 × 10^2^ copies per reaction (line d), and 1.07 × 10^2^ copies per reaction (line e). The virus loads determined in the five spiked human blood samples were as follows: SB1, 1.64 × 10^6^ copies per reaction; SB2, 3.84 × 10^3^ copies per reaction; SB3, 2.17 × 10^3^ copies per reaction; SB4, 6.21 × 10^2^ copies per reaction; and SB5, 1.20 × 10^2^ copies per reaction. NTC, non-template control.
(244 KB DOC).

### Accession Numbers

The GenBank (http://www.ncbi.nlm.nih.gov/Genbank/index.html) accession number for the Frankfurt-1 isolate is AY291315 and for the Urbani isolate is AY278741.

**Patient Summary**

**Background**

Molecular biology (studying the makeup and function of molecules) is increasingly being used to track outbreaks of infectious diseases. For example, molecular biology can help to identify the cause of a disease (such as a virus, bacterium, or parasite) and understand how it spreads.

**Why Was This Study Done?**

These researchers had previously used molecular biology techniques to study different strains of the SARS virus, which causes the often fatal disease called severe acute respiratory syndrome. They had found that different strains could be distinguished from each other on the basis of specific genetic “fingerprints.” They now wanted to find quick and easy ways to determine the identity of particular viral strains found in sick patients.

**What Did the Researchers Do?**

They used a molecular biology technique called mass spectrometry. They took samples from patients (such as blood samples and nasal swabs) and determined whether they could detect the SARS virus, identify specific strains, and distinguish between them.

**What Did They Find?**

They found that mass spectrometry is a useful tool for detecting the SARS virus and for distinguishing between different strains. They also found that they could use this tool to help understand how the SARS virus had been transmitted between specific patients.

**What Are the Limitations?**

For this technique to work, the researchers needed pre-existing information about genetic differences between strains. This means that detailed DNA sequencing is necessary to find these differences in the first place and to discover new ones as the virus evolves.

**What Next?**

The authors suggest that combining initial genetic sequencing of the different strains with the mass spectrometry technique to analyze subsequently large numbers of samples is the most efficient and cost-effective approach.

**More Information Online**

Public access Web pages on SARS from *Science Magazine:* http://www.sciencemag.org/feature/data/sars/

News article on the SARS genome on the Genome Network News Web site: http://www.genomenewsnetwork.org/articles/05_03/sars_3.shtml

Genome Institute of Singapore (GIS): http://www.gis.a-star.edu.sg/homepage/default.jsp

GIS's press release on differences between SARS strains: http://www.gis.a-star.edu.sg/homepage/gismediapress.jsp?pid=19

## Abbreviations

SARS-CoV: severe acute respiratory syndrome coronavirus
MS: mass spectrometry
SARS: severe acute respiratory syndrome
SNV: single nucleotide variation
