# Supplementary figures and images for: SARS Transmission Pattern in Singapore Reassessed by Viral Sequence Variation Analysis
Source: PLoS Med. 2005 Feb 22;2(2):e43. doi: 10.1371/journal.pmed.0020043 (PMC549591; doi:10.1371/journal.pmed.0020043)

Supplement Figure


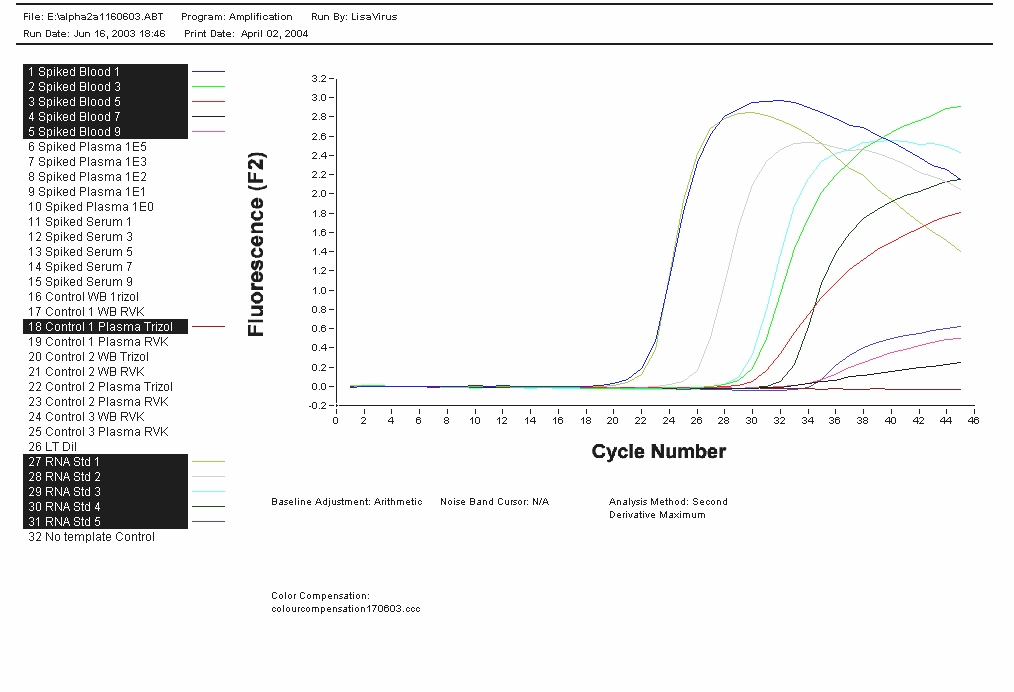


**a**

**b**

**c**

**d**

**e**

**SB1**

**SB2**

**SB3**

**SB4**

**SB5**

**NTC**

Supplement: Figure S1 — The x-axis denotes the cycle number of the quantitative PCR assay, and the y-axis denotes fluorescence intensity (F2) over the background level. RNA standards were as follows: 1.05 × 10^6^ copies per reaction (line a), 1.01 × 10^5^ copies per reaction (line b), 9.4 × 10^3^ copies per reaction (line c), 8.9 × 10^2^ copies per reaction (line d), and 1.07 × 10^2^ copies per reaction (line e). The virus loads determined in the five spiked human blood samples were as follows: SB1, 1.64 × 10^6^ copies per reaction; SB2, 3.84 × 10^3^ copies per reaction; SB3, 2.17 × 10^3^ copies per reaction; SB4, 6.21 × 10^2^ copies per reaction; and SB5, 1.20 × 10^2^ copies per reaction. NTC, non-template control. (244 KB DOC). [file pmed.0020043.sg001.doc]
